# Development of a Modular Sandwich Panel with a Composite Core of Recycled Material for Application in Sustainable Building

**DOI:** 10.3390/polym16243604

**Published:** 2024-12-23

**Authors:** Juan José Valenzuela Expósito, Elena Picazo Camilo, Francisco Antonio Corpas Iglesias

**Affiliations:** Higher Polytechnic School of Linares, University of Jaén, 23700 Linares, Spain; epicazo@ujaen.es (E.P.C.); facorpas@ujaen.es (F.A.C.I.)

**Keywords:** sandwich panel, polymer, modular construction, resin, waste by-products, sustainable materials

## Abstract

In recent years, the construction industry has faced challenges related to rising material costs, labor shortages and environmental sustainability, resulting in an increased interest in modular construction cores composed of recycled materials, such as XPS, PUR, PLW and GFRP, from waste from the truck body industry. Two resins, PUR and polyester, were used to bond these recycled composites. Physical, chemical and mechanical analyses showed that the panels formed with PUR resin had superior workability due to the higher open time of the resin, 11.3% better thermal conductivity than the commercial PLW panel (SP-PLW) and reduced porosity compared to those using polyester resin. The mechanical performance of the panels improved with higher structural reinforcement content (PLW and GFRP). Compared to a commercial panel (SP-PLW), the SP-RCM1 recycled panel showed 4% higher performance, demonstrating its potential for sustainable building applications. Thermal and microscopic characterizations showed good adhesion of the materials in the best performing formulations related to higher thermal stability. Therefore, this research aims to demonstrate the feasibility of using waste from the car industry in the manufacture of sandwich panels for modular construction to address these issues.

## 1. Introduction

The construction industry is positioned as one of the world’s main economic motors, playing a key role in the development of global infrastructure [1,2]. In recent years, construction has faced numerous challenges due to the increased demand for new buildings as a result of rapid population growth, which has put considerable pressure on the sector [3]. In addition, environmental policies have been developed to curb emissions from the manufacture of conventional building materials and contribute to the Sustainable Development Goals (SDGs) proposed by the United Nations (UN) [4,5,6]. On the other hand, increased demand for essential building materials has led to higher material costs. The increase in prices directly affects the economic viability of many projects due to labor shortages associated with the migration of workers to other sectors. These reasons have led to the search for solutions that address the challenges of traditional construction [7].

In response to these challenges, modular construction emerges as an alternative to traditional construction methods due to its ability to meet the challenges of the construction industry [8] due to the reduction of waste and carbon emissions and the reduction in construction times.

This new approach to the construction sector involves the manufacture of panels in a controlled environment that allows for thorough quality control and efficiency in the use of materials. The boom in the application of this construction methodology is focused on various construction sectors such as single-family houses or commercial buildings [9]. Modular manufacturing provides multiple benefits including reduced manufacturing times, reduced associated costs and improvements in environmental impact related to reduced waste generation. Furthermore, off-site manufacturing reduces the risks associated with conventional construction related to the need for lower skilled labor due to greater constructability [10,11,12].

In recent years, many countries have promoted this type of construction due to the versatility in design and construction materials [13,14,15,16]. Modular buildings can be manufactured using a variety of materials, including steel, wood and prefabricated concrete [8,17,18,19]. In the manufacture of modular panels, factors such as light weight are important because the panels must be transported from the manufacturing site to the final location [20]. Transport restrictions due to dimensions and tonnage are postulated as the main disadvantages of modular construction [21] due to land transport regulations and the high weight of the materials requiring specialized equipment for their installation. For this reason, the main objective in this type of construction is to obtain lightweight materials to facilitate transport and reduce costs, while at the same time being resistant and insulating through external reinforcements. This motivates the search for materials that meet these specifications, such as those discussed in this article.

Sandwich panels are widely used in building construction. The reason is that sandwich panels have low thermal conductivity due to their structure and high durability and the manufacturing process is simple. The structure generally consists of a low-density shear-resistant core covered by two outer sheets to provide stiffness. These types of panels are widely used in prefabricated construction due to their high strength-to-weight ratio [22,23] and high flexural strength [24]. The thermal insulation properties of materials such as polyurethane (PUR) or expanded polystyrene (EPS) with low thermal conductivities (0.024–0.030 and 0.035–0.040 W/mK, respectively) [25,26] make these materials optimal lightweight cores that are often combined with GFRP (glass fiber-reinforced polymer) sheets [27]. GFRP coating has advantages over other materials due to its lighter weight, which makes it an alternative for the rehabilitation of old buildings for structural applications [28,29].

Sandwich panel cores can be composed of different materials depending on the final application of the panel; however, cores made of polymeric foams [30] stand out due to their low density and panel cores composed of laminated wood [31] due to their high compressive strength or concrete [12,32,33,34]. Also, the increasing interest in sustainable building practices is encouraging the development of panels with cores composed of sustainable materials or from waste materials from other industries. Waste from the truck body industry can be reused in modular manufacturing due to the high strength-to-weight ratio of the constituent materials and the amount of material generated from the body manufacturing process. Sandwich panel manufacturing is emerging as a viable and sustainable alternative to traditional panels. The use of recycled materials offers multiple environmental and economic benefits focusing on reduced production costs and dependence on virgin raw materials [35,36,37,38,39,40]. From an environmental perspective, the reuse of waste in the manufacture of sandwich panels helps to reduce the amount of waste that ends up in landfills and contributes to increased CO_2_ emissions. By considering the requirements of strength-to-weight ratio, high thermal insulation and shear stiffness, the manufacture of panels incorporating recycled materials is emerging as an alternative to traditional sandwich panels. This trend is in line with the growing interest in sustainable practices and the need to find solutions to the environmental challenges confronting the construction industry.

For all of these reasons, this research focused its attention on the development of modular sandwich panels made of materials from the valorization of waste from the truck body industry. The materials discarded by this industry are mainly composed of wood waste, PUR and extruded polystyrene (XPS) obtained from the leftover material for the shaping of the cores depending on the dimensions of the panel, and GFRP from offcuts from the core cladding. Their use in sandwich panels using virgin materials has been extensively studied, but there is no literature focused on the development of composite cores by material blending. Therefore, the present work aims to investigate the manufacture of sandwich panels using recycled materials such as XPS, PUR, plywood (PLW) and GFRP in combination with commercial resins, which represents an innovative and viable solution to deal with the environmental and economic challenges associated with waste management by reducing raw materials. The thermal insulation and lightweight properties of XPS and PUR, the structural stiffness of PLW and the mechanical reinforcement of GFRP postulate these materials as high value-added materials for modular construction. The use of commercial resins in combination with recycled materials results in high quality and durable products that conform to the standards required for various applications [41,42]. This innovative approach has the potential to transform the construction industry. In this context, the analysis of the adhesion of different resins with recycled material emerges as a topic of great relevance.

Through this article, it has been demonstrated that panels made from recycled materials can achieve the quality and performance standards required for various applications, suggesting a wide potential for their adoption in multiple industries, mainly the construction sector. The integration of recycled materials in panel production is an effective strategy to move towards a circular economy, promoting sustainability.

## 2. Materials and Methods

### 2.1. Raw Materials and Properties

The recycled composite material (RCM) consists of variable proportions of XPS, PUR, PLW and GFRP from truck body manufacturing waste provided by Liderkit (Guarromán, Jaén (Spain)). These materials are obtained as manufacturing waste from offcuts of large panels that are cut to the dimensions specified by each of the products they manufacture. Two different resins were used as binders: 5-1026 polyester resin supplied by Sumarcoop and Neopur 1791 polyurethane resin supplied by Neoflex. Each resin was used with its respective hardener (Promox P200TX and Adiflex 935, respectively). Table 1 shows the technical characteristics of the resins used. The polyester resin has a higher viscosity than the PUR resin (4000 cps (20 °C) and 200–250 cps (25 °C), respectively), which is inversely proportional to the density values (1.20 and 1.62 g/mL, respectively). The manipulation times indicate that, during the open time period, the resins are manipulable in gel format and allow optimal workability for mixing with the RCM material. During the curing time the cores reach 60–70% of the final strength. The curing time represents the total time required to obtain 96–98% of the total strength.

**Figure 1:**
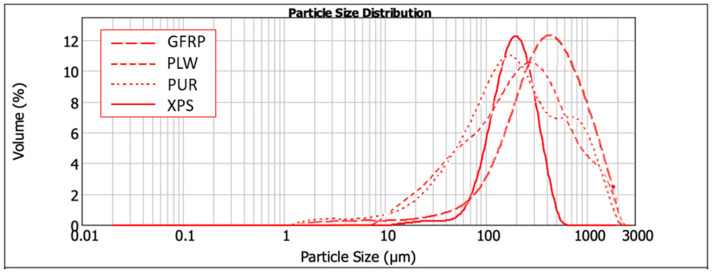
Granulometric distribution of GFRP, PLW, PUR and XPS.

The particle size distribution of the XPS, PUR, PLW and GFRP composing the RCM was determined using the Malvern Mastersizer 2000 (Figure 1). The particles show similar particle sizes as a result of the mechanical recycling of each material in a Felco Europe hammer mill.

**Figure 2:**
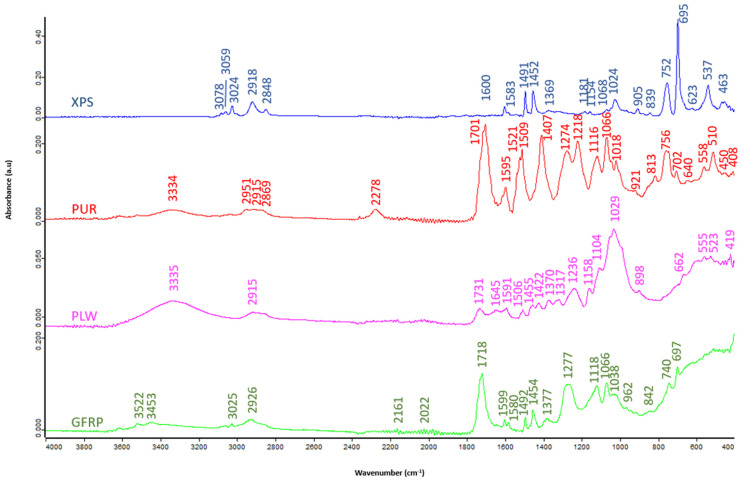
FTIR patterns: XPS, PUR, PLW and GFRP.

The identification of the functional groups was carried out by Fourier transform infrared spectroscopy (FTIR) with the Bruker FT-IR Vertex 70 (Figure 2). The identification range is between 4500 and 600 cm^−1^.

PLW and GFRP show bands appearing at 3335 and 3334 cm^−1^, respectively, associated with O-H stretching vibration [43]. Between 3078 and 2951 cm^−1^, N-H stretching vibration bands appear in GFRP, PUR and XPS typical of amides and characteristic of urethanes [43,44,45]. The bands centered around 2900 cm^−1^ of PLW, PUR and XPS (2915, 2869 and 2918–2848 cm^−1^, respectively) are associated with C-H stretching vibration associated with methyl and methylene groups [44,45]. The bands around 1700 cm^−1^ in PLW and PUR (1731 and 1701 cm^−1^, respectively) correspond to the asymmetric C=O stretching vibration associated with urethanes [45,46]. PLW, GFRP, PUR and XPS show between 1645 and 1218 cm^−1^ vibrational bands of C=C stretching typical of aromatic rings [47,48]. PLW and GFRP show bands associated with asymmetric C-O stretching vibration between 1158–662 cm^−1^ and 1066–697 cm^−1^, respectively [49]. Table 2 shows the characteristic bands for each wavenumber.

The functional groups identified show that the raw materials obtained after mechanical recycling used in the forming of RCM panels are free of contaminants and heavy metals that could interfere with environmental standards and adhesion with the resins used [51,52].

The density of the materials that make up the recycled core (Table 3), calculated by mass and dimensions, is decisive for obtaining lightweight sandwich panels that show good mechanical relationships. The combination of materials with different densities makes it possible to form a panel that combines structural properties (mechanical strength and stiffness) with functional properties (thermal insulation and lightness) [22]. Materials, such as PLW and GRFP, with high densities (505.74 and 1201.67 kg/m^3^, respectively) provide resistance to loads. On the other hand, XPS and PUR present a low density (40.82 and 40.03 kg/m^3^, respectively) due to the cellular structure characteristic of foams where the number of interstitial voids increases. These values are related to improvements in energy efficiency [53].

From the differential thermogravimetric analysis (TG-DSC) carried out by the Metler Toledo analyzer, the thermal stability of the core components was evaluated and information on their behavior under heating conditions was obtained. The test was carried out under heating conditions from ambient temperature up to 500 °C in air atmosphere at a heating rate of 10 °C/min.

The thermal degradation of XPS in air atmosphere shows a mass loss of about 8% related to the breaking of the polymer bonds. The mass loss involves the loss of the structural properties of XPS. Between 350 and 450 °C, the complete decomposition of the polystyrene occurred, and styrene monomers were obtained. At 500 °C, the material had almost completely degraded. The thermal analysis of PUR shows an initial mass loss close to 7% related to the evaporation of the smaller molecules and the decomposition of the urethane groups resulting in the liberation of volatile compounds [54]. The mass loss of 60% at 500 °C implies the loss of structural properties of PUR. Regarding PLW, the material starts thermal degradation near 200 °C but most of the degradation occurs between 250 and 400 °C, which is typical of lignocellulosic materials. The endothermic peak observed at 336 °C corresponds with the thermal degradation of cellulose and other organic components such as lignin and hemicellulose [55]. The mass loss of GFRP is lower than the other materials; therefore, the thermal stability is higher. The degradation of this material starts at 241 °C, and at 500 °C, the material only shows a mass loss of 37%.

**Figure 3:**
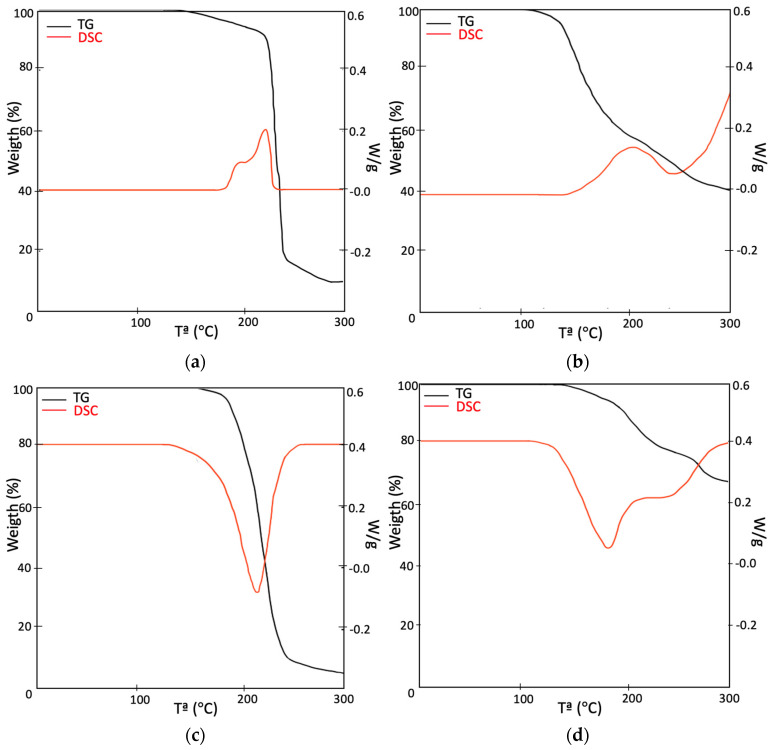
TG-DSC: (**a**) XPS, (**b**) PUR, (**c**) PLW and (**d**) GFRP.

Figure 3 shows the TG and DSC curves of PUR, XPS, PLW and GFRP carried out in air atmosphere up to 500 °C at a heating rate of 10 °C/min. Table 4 provides the parameters obtained from the test results.

### 2.2. Conformation and Characterization of Panels

Experimentally, a total of 10 families, each consisting of 10 specimens (SP-RCM), were developed and compared with a commercial PLW composite core panel (SP-PLW). All core families were prepared with varying proportions of PUR (0.50, 2.25, 3.75 and 5.00%w), XPS (0.50, 2.25, 3.75 and 5.00%w), PLW (32.00, 30.00, 29.00, 28.00 and 27.00%w) and GFRP (32.00, 30.50, 28.50, 2.00 and 25.50%w). Two types of resins were used as binder: Neopur 1791 PUR resin (26.25%w) (SP-RCM1–SP-RCM5) and 5-1026 polyester resin (34.30%w) (SP-RCM6–SP-RCM10), each with a corresponding hardener (8.75 and 0.70%w, respectively) (Table 5). The RCM/resin ratio remained constant (1.9) in all of the families developed. All of the tested panels had dimensions of 20 × 20 × 2 cm and the GFRP plates covering the cores had a thickness of 1.4 mm.

**Figure 4:**
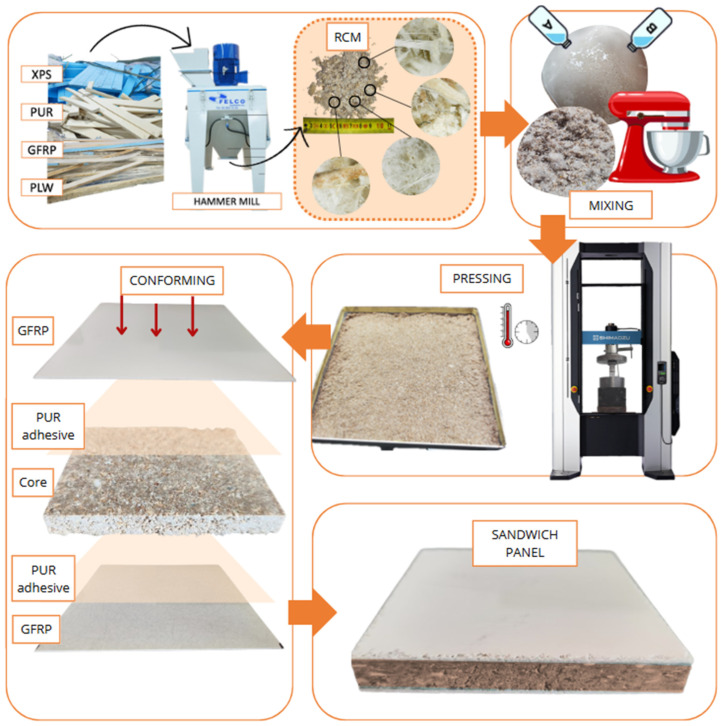
SP-RCM manufacturing process (A: resin, B: hardening agent).

The binder was prepared by mixing the corresponding amounts of resin and hardening agent until a homogeneous mixture was obtained. The cores were obtained by mixing the binder with RCM in the proportions defined in Table 3 in a Proeti planetary mixer. The resulting mixture was poured into a mold measuring 20 × 20 × 2 cm and pressed at 15 MPa in a Shimadzu AG-300 KNX press, with the load being maintained for 6 h at a controlled temperature of 25 ± 2 °C. After the total curing time (24 h), the cores were polished until a flat surface was obtained to facilitate the adhesion of the glass fiber reinforced polyester (GFRP) sheets on the outer faces to obtain the sandwich panels. The adhesion of both GFRP panels was carried out using a PUR Neopur 1791 adhesive in a 75:25 adhesive/hardener ratio. The first 1.4 cm plate was applied uniformly with 165 g/m^2^ of adhesive and the core was placed on top of the first plate. The second plate, of the same thickness as the first, is adhered by pouring the adhesive over the core and then placing the GFRP plate on top. Then, for a good adhesion of the GFRP plates, a pressure of 1.5 MPa was applied to the sandwich panel and maintained for 6 h. After this time, the panels remained at a controlled temperature of 25 ± 2 °C for 48 h. The curing process was the same for both resins. Figure 4 schematically represents the process developed for the forming of the panels.

The properties of sandwich panels are studied by physical, chemical, mechanical and microscopic tests detailed in Table 6 under the corresponding standards.

Moisture absorption is directly related to dimensional stability. Both parameters study the behavior of sandwich panels under the influence of moisture [63]. Both tests were performed in a Dycometal SSC 140 humidity chamber under the respective standards shown in Table 6. The importance of the porosity determination is focused on the evaluation of how the porous cavities affect the structural parameters. The number of pore cavities was obtained by X-ray computed tomography test on the SkyScan 2214 Bruker (pixel size 1.95 µm). The pore index influences the density values and consequently the strength-to-weight ratio, affecting other parameters such as compressive strength and flexural strength [41] which were determined with the Shimadzu AG-300 KNX equipment. For the determination of the compressive strength, specimens measuring 100 × 100 × 16 mm were tested and placed between the plates of the equipment and a preload of 250 Pa was applied. The specimen was then compressed until failure. The determination of the bending strength was carried out on 300 × 20 × 15 mm specimens biaxially braced with a span of 240 mm. The density was calculated by dimensional and mass determination. On the other hand, the cavity volume is directly related to the degree of insulation of the sandwich panels. The thermal conductivity was evaluated by means of the HFM 446 Lambda Eco-Line Netzsch. Panels with dimensions 20 × 20 × 4 cm were tested in the equipment consisting of dual heat flow translators. The equipment was previously calibrated with reference materials of known thermal conductivity.

The thermal test performed by TG-DSC on Metler Toledo equipment was used to study the curing behavior of adhesives in air atmosphere and the influence of temperature on the mass loss of raw materials that influence the structure [64]. For the test, 10 × 10 × 10 mm specimens were introduced into the previously calibrated equipment. Other authors propose the measurement of thermal properties using thermal chambers or sensors [65,66,67].

Chemical compatibility was carried out to study the compatibility between the resins and the raw materials used due to the differences in the polarity of the materials that can produce adhesion problems depending on the thermodynamic affinities [68]. The test was performed by FTIR on the Vertex 70 Bruker.

Finally, the microscopic analysis of fragments of the panels by scanning electron microscopy (SEM) and energy dispersive X-ray spectroscopy (EDX) allowed the microstructure to be evaluated optically and chemically.

## 3. Results

### 3.1. Physical Characterization of Panels

Table 7 shows the results obtained from the physical characterization of the different formed panels after 48 h at a controlled temperature of 25 ± 2 °C.

**Figure 5:**
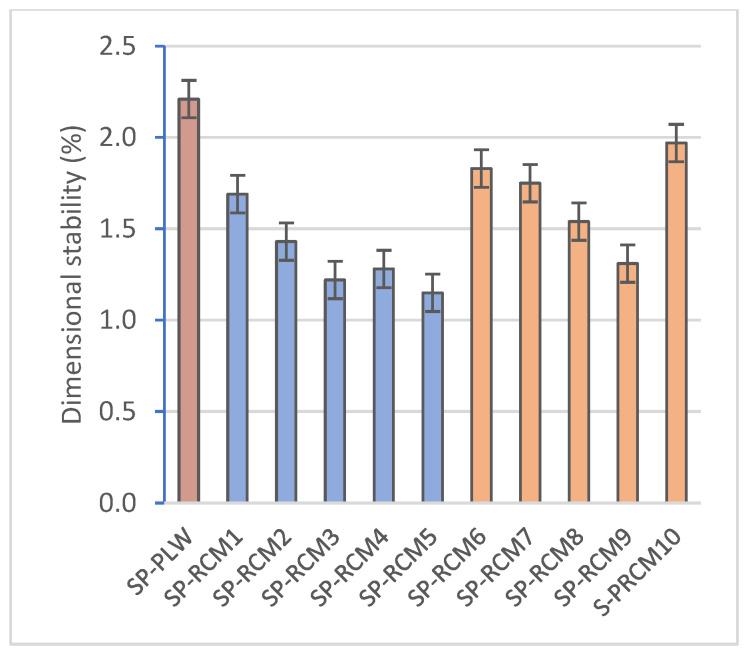
Dimensional stability (%) of SP-PLW and SP-RCM panels.

The results obtained for dimensional stability (Figure 5) show insignificant values (less than 2%) and are mainly associated with the evaporation of free water from the resins used. The materials that make up the RCM mix show a low tendency to shrinkage. XPS and PUR have a closed cell structure that provides stability during the curing process. On the other hand, PLW, despite being a porous material, has a low shrinkage tendency that is favored by the orientation of its wood layers [69], which is reflected in the SP-PLW value obtained (2.21%). GFRP acts as a reinforcement in the core and strengthens the panel by sandwiching the external boards.

**Figure 6:**
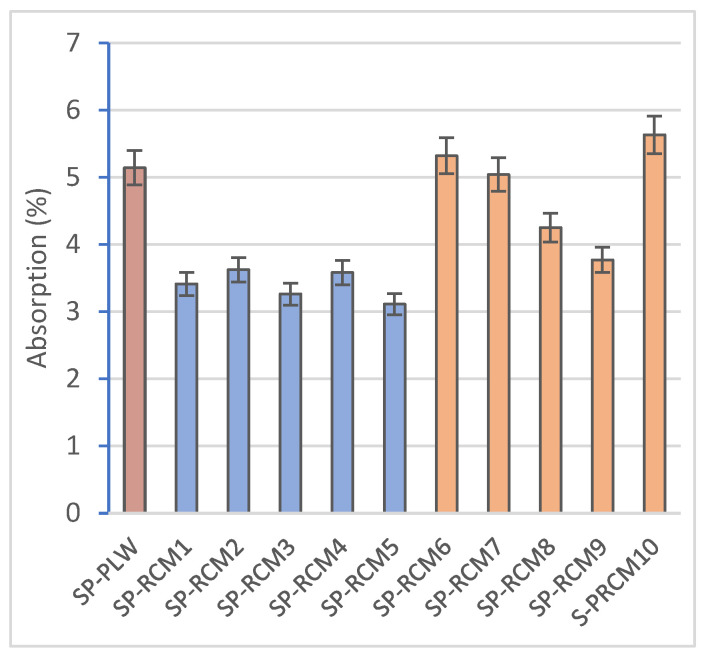
Absorption (%) of SP-PLW and SP-RCM panels.

**Figure 7:**
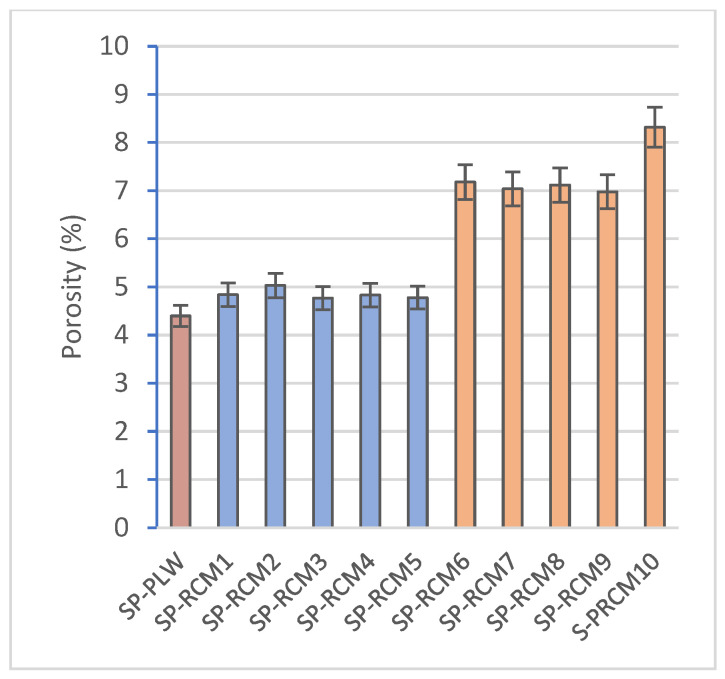
Porosity (%) of SP-PLW and SP-RCM panels.

**Figure 8:**
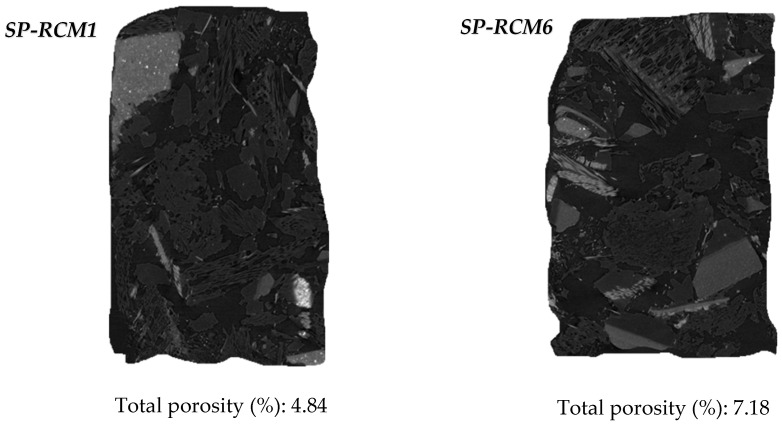
Nanocomputed images (1.95 µm) of SP-RCM1 and SP-RCM6.

The absorption values (Figure 6), associated with high dimensional stability and porosity, are reduced compared to SP-PLW (5.14%) and do not show variations between panels formed with different proportions of PUR, XPS, PLW and GFRP. However, higher absorption values (between 3.77 and 5.63%) are observed for panels containing polyester resin (SP-RCM6–SP-RCM10) compared to those formed with PUR resin (SP-RCM1–SP-RCM5), which are close to 3%. This increase is due to the workability of the polyester resin which, despite having less viscosity than the PUR resin, shows a worse workability and the open time is reduced (Table 1). The worse workability results in inhomogeneous mixing with the RCM material and unimpregnated particles due to the lower sealing capacity. The pore volume of the RCM sandwich panels is influenced by the mixing and forming process of the mixtures and the working times of the resins. The porosity values (Figure 7) of SP-RCM1–SP-RCM5 of around 5% verify the better workability and mixing of the PUR resin with the RCM material. In contrast, the SP-RCM6–SP-RCM9 panels show values around 7% porosity. SP-RCM10 exhibited the highest percentage of porosity (8.32%). The observed increase is associated with the problems associated with the difficulties in handling the polyester resin and the reaction that occurs between the polyester resin and the PUR contained (6.25%) in the RCM. The reaction causes adhesion problems associated with the thermodynamic affinities of the materials [70]. Figure 8 shows X-ray computed tomography images of SP-RCM1 and SP-RCM6.

**Figure 9:**
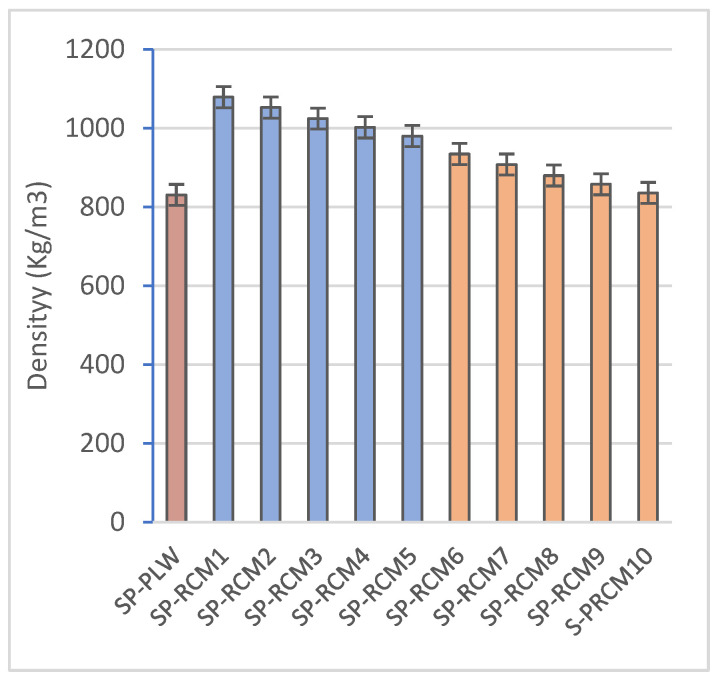
Density (kg/m^3^) of SP-PLW and SP-RCM panels.

The density of the panels (Figure 9) is mainly influenced by the weight percentages of the constituent materials of the RCM material, as the porosity does not have a significant influence as it remains almost constant between panels formed with the same resin. In the panels formed with PUR resin, the mixtures with the highest content of PLW and GFRP (both 32.0%) and the lowest content of PUR and XPS (both 0.5%) (SP-RCM1) show the highest density (1078.78 kg/m^3^) while the SP-RCM5 panel, with the lowest content of PLW and GFRP (27.0 and 25.5%), and the panel with the lowest content of PUR and XPS (both 6.25%) (SP-RCM1) show the highest density (1078.78 kg/m^3^). This is associated with the densities of each of the raw materials used (Table 3). Similarly, the sandwich panels formed with PUR resin followed the same trend showing the highest density for SP-RCM6 (934.58 kg/m^3^) and the lowest density for SP-RCM10 (835.84 kg/m^3^). On the other hand, the panels containing polyester resin have higher densities than those made with PUR resin as a consequence of the difference in density (1109 and 1620 kg/m^3^, respectively). The SP-PLW control panel presented a lower density (830.65 kg/m^3^) as a consequence of the lower resin content due to the fact that the material is composed of thin layers of wood glued with thin layers of resin and subjected to pressure.

**Figure 10:**
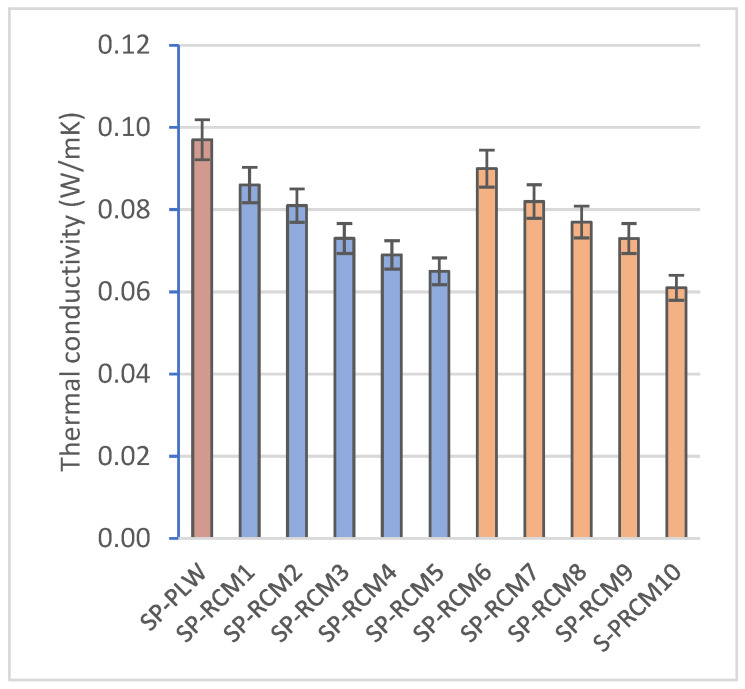
Thermal conductivity (W/mK) of SP-PLW and SP-RCM panels.

The thermal conductivity values of sandwich panels (Figure 10) are associated with the thermal properties of the constituent materials, with the interaction of these materials in the core and with the thickness. PUR, XPS and PLW have values of 0.024, 0.032 and 0.1 W/mK, respectively, while the thermal conductivity of GFRP is considered negligible. On the other hand, PUR resin has a lower thermal conductivity than polyester resin (0.027 and 0.21 W/mK). The results obtained after the evaluation of the panels confirm this. The panels that were formed with PUR resin showed a higher thermal resistivity, obtaining values between 0.065 (SP-RCM5) and 0.086 W/mK (SP-RCM1). In contrast, the polyester resin composite panels showed significantly higher thermal conductivity values (between 0.134 (SP-RCM6) and 0.097 W/mK (SP-RCM9)). The decrease in the thermal resistivity of SP-RCM10 is associated with the increased porosity of the panel due to the aforementioned problems. On the other hand, the conductivity value of SP-PLW (0.097 W/mK) is higher than those of SP-RCM panels as a consequence of the constituent material and the lower porosity of the material.

### 3.2. Chemical Characterization of Panels

**Figure 11:**
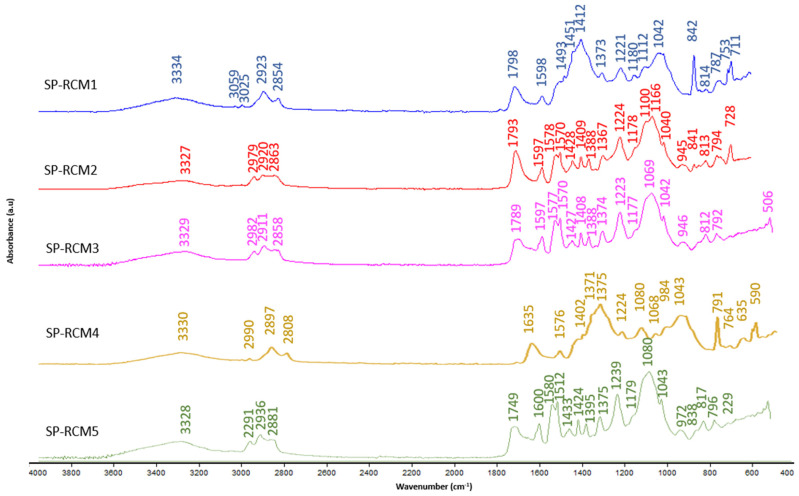
FTIR patterns: SP-RCM1–SP-RCM5.

**Figure 12:**
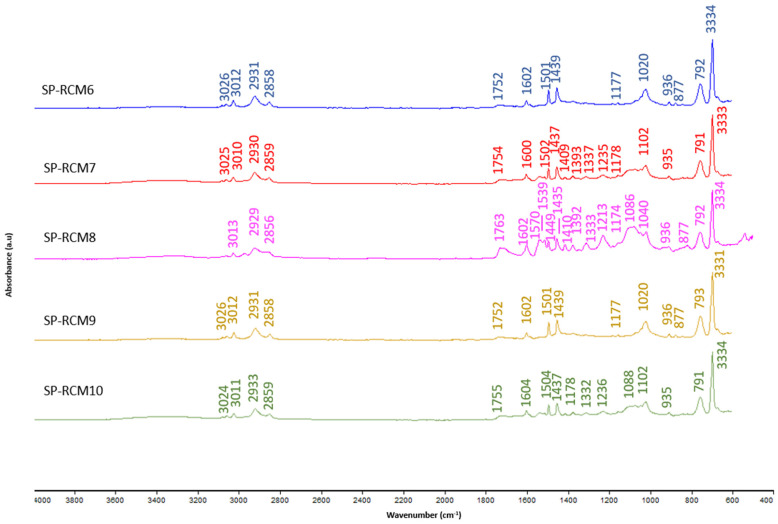
FTIR patterns: SP-RCM6–SP-RCM10.

The FTIR spectra of the cores of the panels SP-RCM formed were analyzed after the end of the curing time to identify the functional groups resulting from the reactions produced between the raw materials and the resins used. Figure 11 shows the FTIR spectra of the SP-RCM1–SP-RCM5 panels formed with PUR resin and Figure 12 shows the FTIR spectra of the SP-RCM6–SP-RCM10 panels. The bands appearing between 3334 and 3011 cm^−1^ suggest the presence of O-H bonds related to PLW water adsorption [43,70]. In the range between 2808 and 2936 cm^−1^ appear C-H stretching vibration bands characteristic of organic compounds such as polymers [70,71]. Asymmetric C=O stretching vibrations in the range 1798–1600 cm^−1^ are associated with the presence of PUR and polyester resins [68]. The bands appearing in the range 1597–1221 cm^−1^ correspond to the C=C stretching vibration typical of aromatic rings in building materials [72]. The C-O bands produced by the asymmetric stretching vibration located between 1180 and 1020 cm^−1^ are associated with the presence of PUR resin in the SP-RCM1–SP-RCM5 matrices and polyester resin in the SP-RCM6 and SP-RCM10 matrices [50,71]. Finally, the vibration of aromatic groups between 972 and 711 cm^−1^ suggests the presence of C-H bonds in the polymeric components [72]. Table 8 shows the characteristic groups for each wavenumber.

### 3.3. Mechanical Characterization of Panels

**Figure 13:**
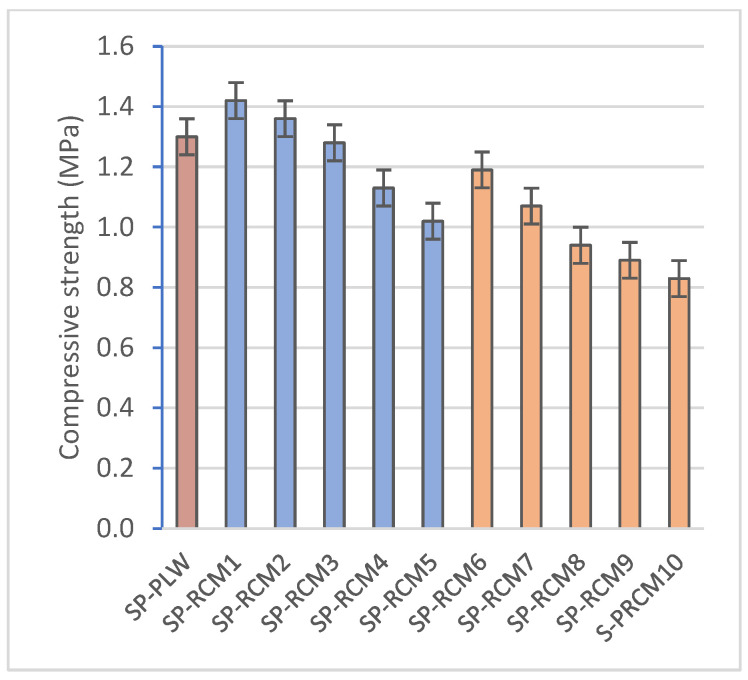
Compressive strength (MPa) of SP-PLW and SP-RCM panels.

**Figure 14:**
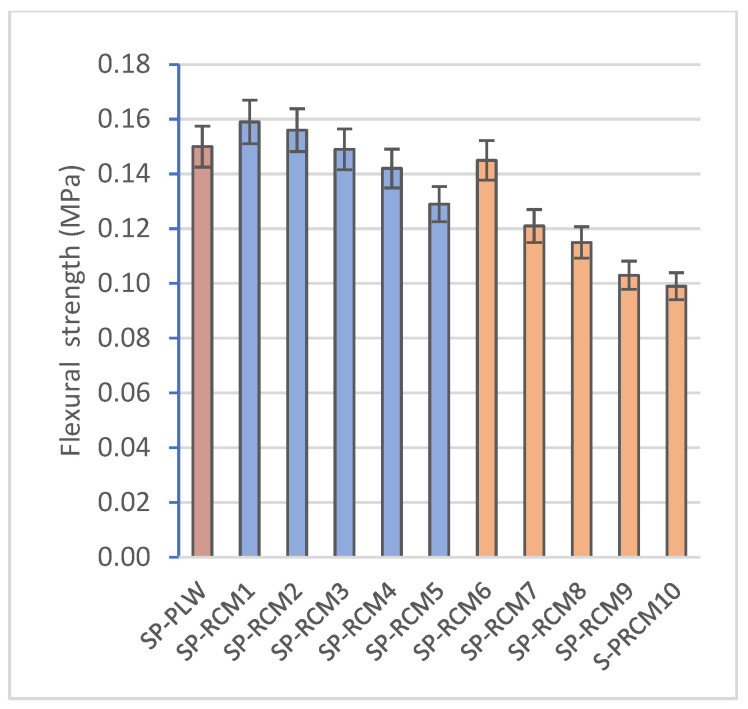
Flexural strength (MPa) of SP-PLW and SP-RCM panels.

Table 9 shows the experimental results of the compressive and flexural strength tests of each of the sandwich panels formed after 72 h of curing at a controlled temperature of 25 ± 2 °C and of the commercial SP-PLW panel. The results obtained show that the composition of the sandwich panels influences the mechanical strength. In SP-RCM1 and SP-RCM6, the increase in the proportions of GFRP (32%) and PLW (26.25%) increases the values of compressive strength (1.42 and 1.19 MPa, respectively) (Figure 13) and flexural strength (0.159 and 0.145 MPa, respectively) (Figure 14) associated with the densification of the constituent materials. SP-RCM1 showed higher flexural and compressive strength values than the commercial SP-PLW panel. On the other hand, it is observed how resin influences the mechanical characteristics. The panels formed with PUR resin (SP-RCM1–SP-RCM5) showed slightly higher strengths than those obtained in the panels formed with polyester resin (SP-RCM6–SP-RCM10). This is associated with the poor impregnation of the RCM material with the polyester resin due to the workability of the resin as discussed above. SP-RCM8, SP-RCM9 and SP-RCM10 show the lowest compressive (0.94, 0.89 and 0.83 MPa, respectively) and flexural strengths (0.115, 0.103 and 0.099 MPa, respectively) due to the reaction produced between the resin and the PUR that constitutes the matrix, which prevents a good cohesion of the materials.

### 3.4. Thermal Characterization of Panels

The TG-DSC analysis was carried out to evaluate the thermal stability of the SP-RCM1 and SP-RCM6 sandwich panels, which showed the best results for mechanical resistance, and the performance of each of the resins. The performance was evaluated in an air atmosphere with a flow rate of 50 mL/min at a heating rate of 10 °C/min in the temperature range of 30 to 300 °C.

**Figure 15:**
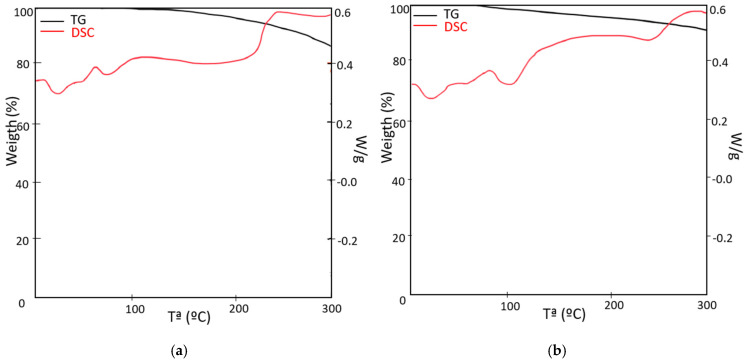
Curves TG-DSC: (**a**) SP-RCM1 and (**b**) SP-RCM6.

The thermogravimetric analysis of SP-RCM1 (Figure 15a) shows the mass variation experienced by the material as a function of temperature. The TG-DSC curve shows a first interval (between 28.65 and 122 °C) that presents a mass loss of 1.33%. This loss is associated with the evaporation of the water absorbed by the porous materials of the core or the solvents present in the panel components and a glass transition of the polymeric components that constitute the sandwich panel. In the second range (between 123 and 297 °C), the mass loss is more significant (8.65%). The loss is directly related to the thermal degradation observed in the endothermic peak (237 °C) suffered by the polymers that constitute the sandwich panel. In this region, PUR, XPS and GFRP suffered a decomposition process. The thermogravimetric analysis of the SP-RCM6 panel (Figure 15b) showed similar behavior. The TG curve shows two intervals, the first one presents a mass loss of 1.48% between 27.33 and 116 °C associated with the evaporation of the most volatile components (water and solvents), while the second interval between 117 and 293 °C shows a mass loss of 9.50% that coincides with the decomposition of the core polymers. Table 10 gives the parameters obtained from the test results.

### 3.5. Microscopic Characterization of Panels

**Figure 16:**
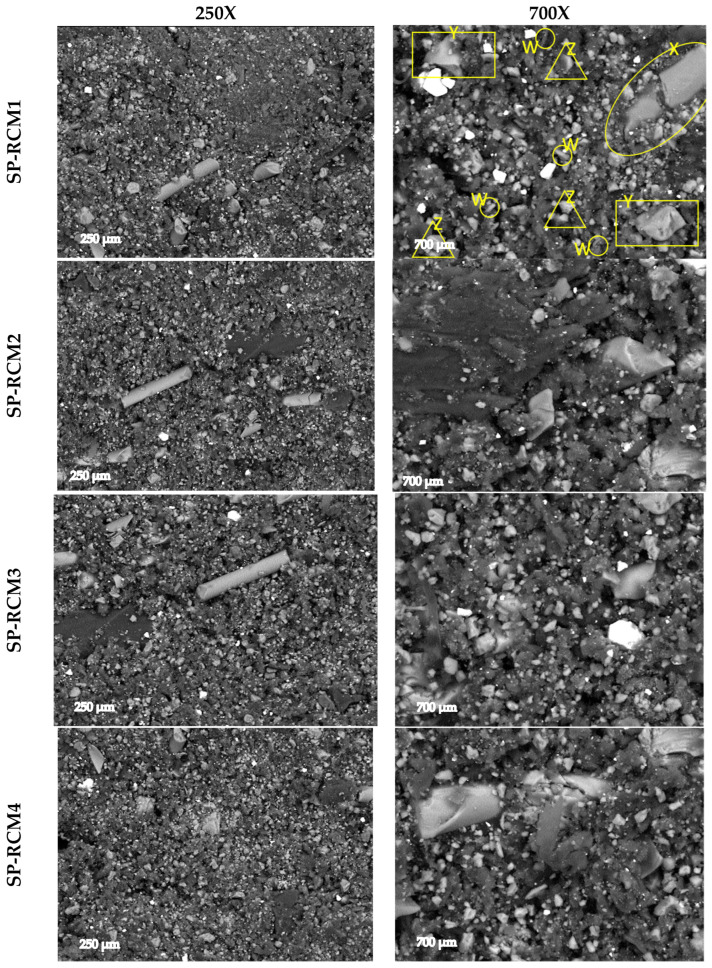

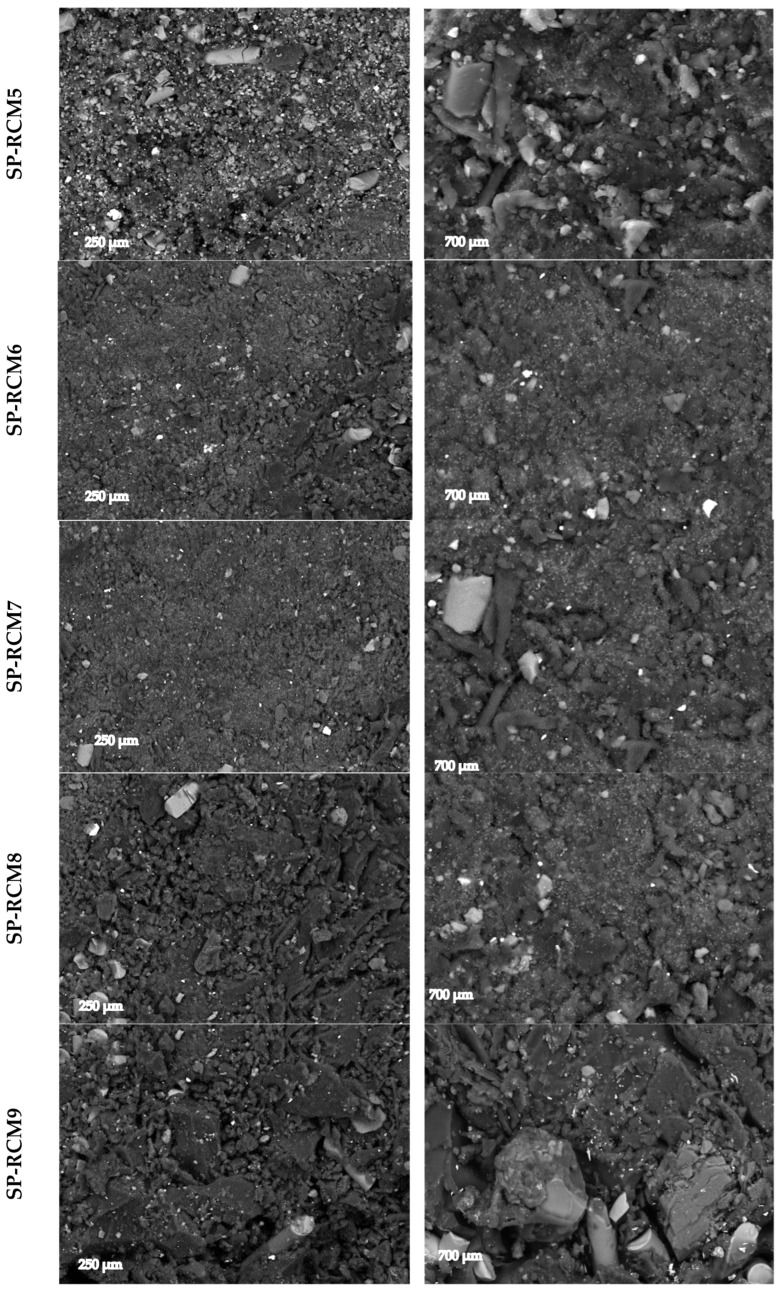

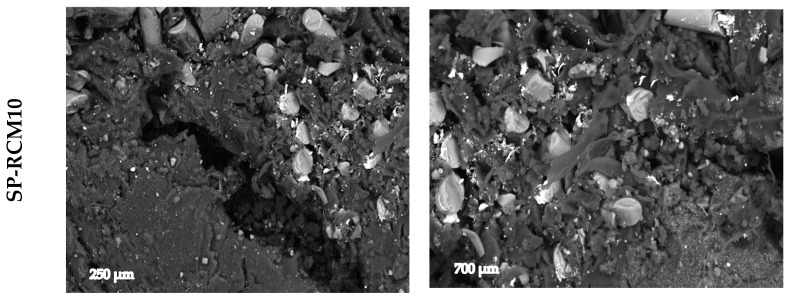
SEM images of SP-PLW and SP-RCM panels at 250X and 700X.

All SP-RCM panels formed with GFRP (X), PUR (Y), XPS (Z) and PLW (W) and PUR and polyester resins were analyzed by SEM after 72 h of curing at room temperature (Figure 16). The bonding of the interfaces of the core materials was optically analyzed to evaluate the adhesion. The correct adhesion of the materials is related to improvements in thermal stability due to better thermal distributions. SP-RCM10 shows a less densified structure as a consequence of the chemical incompatibility of the polyester resin and the XPS. The reaction produced between the polystyrene contained in the XPS and the styrene present in the polyester resin produces solvent attacks that result in a weakening of the XPS structure and a lack of cohesion and loss of mechanical properties. SP-RCM1 and SP-RCM6 show the densest and most uniform structures (700 µm) related to a better integration of the core materials. In all panels, elongated fibers are observed corresponding to GFRP present in the cores. SP-RCM10 shows a higher degree of GFRP agglomeration due to workability problems of the RCM mix and polyester resin and the degradation of other components such as XPS.

**Figure 17:**
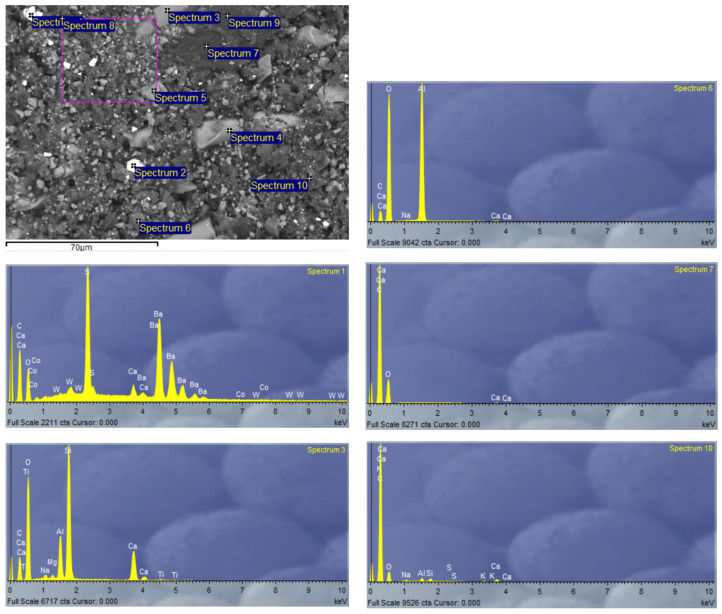
EDX analysis of SP-RCM1.

**Figure 18:**
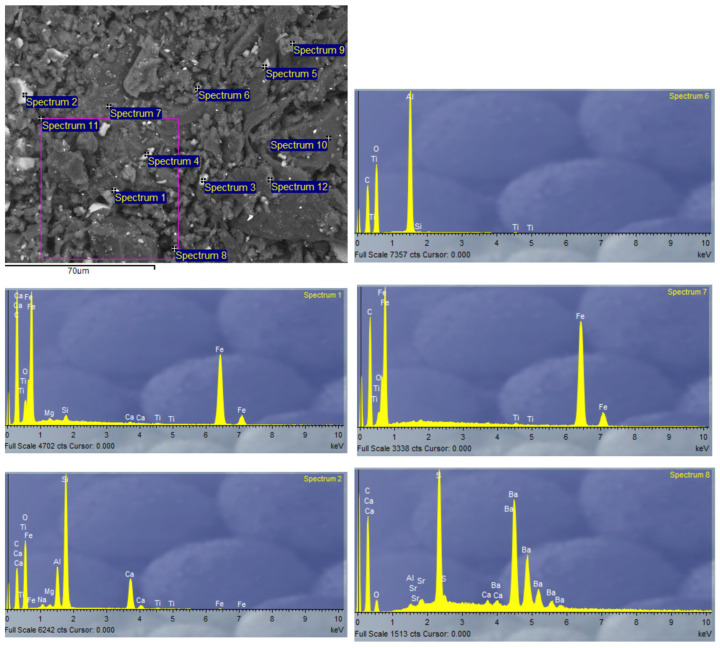
EDX analysis of SP-RCM6.

Due to the heterogeneity of the constituent materials of the panels, EDX analysis was performed on SP-RCM1 and SP-RCM6 to analyze the morphology. Figure 17 and Figure 18 show high concentrations of carbon (C) and oxygen (O) characteristic of the polymers (PUR and polyester resin) and organic matter present in PLW. The presence of calcium (Ca) and silicon (Si) is related to the inorganic components of the reinforcements in the GFRP layers because this material is mainly composed of SiO_2_ and CaO. On the other hand, the presence of barium is related to the additives present in PLW coatings.

## 4. Conclusions

This study evaluates the development of a modular sandwich panel formed with recycled materials from the truck body industry by studying the physical, chemical and structural properties of the developed panels. The following conclusions are drawn:

- The study confirms the feasibility of using recycled materials (PLW, GFRP, PUR and XPS) in varying proportions for forming sandwich panel cores, with PUR and polyester resins showing promising results for mechanical and thermal stability.
- The thermal conductivity of SP-RCM panels remains within building material standards (0.065–0.090 W/mK) and correlates with porosity. Panels with PUR resin (SP-RCM1–SP-RCM5) showed consistent porosity (~5%), while those with polyester resin (SP-RCM6–SP-RCM10) had increased porosity, peaking at 8.32% in SP-RCM10, due to adhesion issues. SP-RCM panels showed 7–37% improvements in conductivity over SP-PLW.
- All panels showed water absorption ratios between 3 and 5%, but SP-RCM10 (5.63%) and SP-PLW (5.14%) had higher absorption due to lower material adhesion caused by thermodynamic affinity problems of the polyester resin and PUR. SP-RCM1 showed 33.7% lower absorption compared to SP-PLW.
- The increase in PLW and GFRP content in the core (SP-RCM1 and SP-RCM6) shows a significant increase in the mechanical properties of the sandwich panels, obtaining compressive strength values of 1.42 and 1.19 MPa, respectively, and flexural strength values of 0.159 and 0.145 MPa, respectively, while SP-PLW presented intermediate values (1.30 and 0.150 MPa, respectively). Improvements of 4% were obtained in SP-RCM1 compared to SP-PLW. This increase is associated with the increase in the core density of SP-RCM1 (1078.78 kg/m^3^) and SP-RCM6 (934.58 kg/m^3^) due to the structural reinforcement provided by PLW, related to the lignocellulosic structure and the laminated structure of the material, and by GFRP related to the glass fibers and the polymeric matrix.
- TG-DSC analysis showed superior thermal stability in SP-RCM1, with slower degradation and gradual mass loss compared to SP-RCM6.
- SEM images revealed good resin adhesion and low porosity in most panels, but SP-RCM10 exhibited adhesion failures and higher porosity due to material workability issues with polyester resin.
- PUR resin is postulated as the most efficient resin because it presents better workability and handling times are longer than those of the polyester resin. On the other hand, the PUR resin did not present problems of thermodynamic affinities with the materials that compose the RCM material [51,73].
- The development of the sandwich panel with a composite core of recycled material is feasible for industrial scale-up by mechanically recycling the waste and using specialized machinery to form the panels.
- These panels align with the circular economy model, offering a sustainable solution for modular construction. Panels with higher PUR and GFRP content exhibit reduced thermal conductivity and mechanical strength suitable for lightweight structural applications, ensuring compliance with technical and environmental standards.

Based on these conclusions, it can be stated that the use of recycled material from truck bodies (PLW, GFRP, PUR and XPS) for sandwich panels for use in modular construction is feasible and can compete with commercial PLW panels. By varying the core composition and resin used, the physical and mechanical properties can be optimized.

## Figures and Tables

**Table 1:** Technical parameters polyester resin 5-1026 and Neopur 1791.

Resin Type	Curing Temperature (°C)	Time Types	Waiting Times (min)	Viscosity (cps)	Density (kg/m^3^)
Polyester resin 5-1026	25	Open time	16–20		
		Hardening time	32–37	4000 (20 °C)	1109
		Curing time	1440		
Neopur 1791	20–22 °C	Open time	40–45		
		Hardening time	300–480	200–250 (25 °C)	1620
		Curing time	1440		

**Table 2:** Characteristic absorption peaks of FTIR spectra of PLW, PUR, XPS and GFRP.

Functional Group	Wavenumber Range (cm^−1^)	FTIR Peaks (cm^−1^)				References
Raw Materials		PUR	XPS	PLW	GFRP	
Stretching vibration O-H	3335, 3334	3334	-	3335	-	[43,44]
Stretching vibration N-H	3078–2951	2951	3078–3024	-	3025	[44,45,46]
Stretching vibration C-H	2915–2848	2869	2918–2848	2915	-	[44,45]
Asymmetric stretching vibration C=O	1731–1701	1701	-	1731	1718	[46,47]
Stretching vibration C=C	1645–1218	1595–1274	1600–1349	1645–1236	1599–1277	[48,49]
Asymmetric stretching vibration C-O	1158–662	1116–640	1181–695	1158–662	1066–697	[43,44,50]

**Table 3:** Density of XPS, PUR, PLW and GFRP.

Raw Materials	Density (kg/m^3^)
XPS	40.82
PUR	40.03
PLW	505.74
GFRP	1201.67

**Table 4:** Parameters obtained from TG-DSC results for thermal degradation of XPS, PUR, PLW and GFRP.

Atmosphere	Raw Material	Temperature Range (°C)	Weight Loss (%)
Air	XPS	0–365	7
		365–400	84
		400–500	92
	PUR	0–220	8
		220–440	51
		440–500	60
	PLW	0–337	3
		337–398	88
		398–500	94
	GFRP	0–302	11
		302–420	28
		420–500	37

**Table 5:** Proportions of SP-PLW and SP-RCM panels.

Specimen	PUR (%)	XPS (%)	PLW (%)	GFRP (%)	PUR Resin (%)	Polyester Resin (%)	Hardening Agent (%)	RCM/Resin
SP-PLW	-	-	100	-	-	-	-	-
SP-RCM1	0.50	0.50	32.00	32.00	26.25	-	8.75	1.9
SP-RCM2	2.25	2.25	30.00	30.50	26.25	-	8.75	1.9
SP-RCM3	3.75	3.75	29.00	28.50	26.25	-	8.75	1.9
SP-RCM4	5.00	5.00	28.00	27.00	26.25	-	8.75	1.9
SP-RCM5	6.25	6.25	27.00	25.50	26.25	-	8.75	1.9
SP-RCM6	0.50	0.50	32.00	32.00	-	34.30	0.70	1.9
SP-RCM7	2.25	2.25	30.00	30.50	-	34.30	0.70	1.9
SP-RCM8	3.75	3.75	29.00	28.50	-	34.30	0.70	1.9
SP-RCM9	5.00	5.00	28.00	27.00	-	34.30	0.70	1.9
SP-RCM10	6.25	6.25	27.00	25.50	-	34.30	0.70	1.9

**Table 6:** Parameters, standards and equipment used.

Parameter	Standard	Equipment
Moisture absorption	UNE-EN 60068-2-67-1997/A1:2019 [56]	Moisture chamber Dycometal SSC 140
Dimensional stability	UNE-EN 1604:2013 [57]	Moisture chamber Dycometal SSC 140
Thermal conductivity	UNE-EN 12667:2002 [58]	HFM 446 Lambda Eco-Line Netzsch
Density	UNE-EN ISO 29470:2021 [59]	Balance RB-30KG Cobos
Porosity	-	SkyScan 2214 Bruker
Compressive strength	UNE-EN 826:2013 [60]	Shimadzu AG-300 KNX
Flexural strength	UNE-EN ISO 141251999-A1:2011 [61]	Shimadzu AG-300 KNX
TG-DTG-DSC	UNE-EN ISO 11357-1:2023 [62]	Metler Toledo
FTIR	-	FT-IR Vertex 70 Bruker
SEM-EDX	-	Microscope Carl Zeiss Merlin

**Table 7:** Results of physical parameters of SP-PLW and SP-RCM panels.

Specimen	Dimensional Stability (%)	Absorption (%)	Density (kg/m^3^)	Porosity (%)	Thermal Conductivity (W/mK)
SP-PLW	2.21 ± 0.12	5.14 ± 0.38	830.65 ± 21.42	4.40 ± 0.11	0.097 ± 0.004
SP-RCM1	1.69 ± 0.31	3.41 ± 0.54	1078.78 ± 25.46	4.84 ± 0.13	0.086 ± 0.005
SP-RCM2	1.43 ± 0.22	3.62 ± 0.39	1052.05 ± 38.94	5.03 ± 0.24	0.081 ± 0.005
SP-RCM3	1.22 ± 0.24	3.26 ± 0.18	1024.17 ± 32.11	4.77 ± 0.15	0.073 ± 0.002
SP-RCM4	1.28 ± 0.13	3.58 ± 0.38	1002.10 ± 30.70	4.83 ± 0.24	0.069 ± 0.004
SP-RCM5	1.15 ± 0.15	3.11 ± 0.45	980.03 ± 32.19	4.78 ± 0.19	0.065 ± 0.008
SP-RCM6	1.83 ± 0.18	5.32 ± 0.30	934.58 ± 27.69	7.18 ± 0.14	0.090 ± 0.004
SP-RCM7	1.75 ± 0.25	5.04 ± 0.19	907.86 ± 19.81	7.04 ± 0.20	0.082 ± 0.003
SP-RCM8	1.54 ± 0.11	4.25 ± 0.22	879.98 ± 13.78	7.12 ± 0.17	0.077 ± 0.004
SP-RCM9	1.31 ± 0.09	3.77 ± 0.43	857.91 ± 21.13	6.98 ± 0.09	0.073 ± 0.007
SP-RCM10	1.97 ± 0.15	5.63 ± 0.37	835.84 ± 25.58	8.32 ± 0.22	0.061 ± 0.006

**Table 8:** Characteristic absorption peaks of FTIR spectra of SP-RCM panels.

Functional Group	Wavenumber Range (cm^−1^)	References
Stretching vibration O-H	3334–3011	[43,70]
Stretching vibration C-H	2936–2808	[70,71]
Asymmetric stretching vibration C=O	1798–1635	[71]
Stretching vibration C=C	1597–1221	[72]
Asymmetric stretching vibration C-O	1180–1043	[50,72]
Stretching vibration C-H	972–711	[72]

**Table 9:** Results of mechanical parameters of SP-PLW and SP-RCM panels.

Specimen	Compressive Strength (MPa)	Flexural Strength (MPa)
SP-PLW	1.30 ± 0.15	0.150 ± 0.015
SP-RCM1	1.42 ± 0.12	0.159 ± 0.009
SP-RCM2	1.36 ± 0.09	0.156 ± 0.005
SP-RCM3	1.28 ± 0.15	0.149 ± 0.006
SP-RCM4	1.13 ± 0.06	0.142 ± 0.003
SP-RCM5	1.02 ± 0.07	0.129 ± 0.011
SP-RCM6	1.19 ± 0.08	0.145 ± 0.004
SP-RCM7	1.07 ± 0.12	0.121 ± 0.002
SP-RCM8	0.94 ± 0.08	0.115 ± 0.004
SP-RCM9	0.89 ± 0.09	0.103 ± 0.008
SP-RCM10	0.63 ± 0.08	0.081 ± 0.006

**Table 10:** Parameters obtained from TG-DSC results for thermal degradation of SP-RCM1 and SP-RCM6.

Atmosphere	Sample	Temperature Range (°C)	Weight Loss (%)	Heat (J/g)
Air	SP-RCM1	0–122	1.33	−25.56
		123–297	8.65	39.06
	SP-RCM6	0–116	1.48	−23.09
		117–293	9.50	36.31

## Data Availability

Data are contained within the article.
